# Glucocorticoid receptor-regulated *TcLEC2* expression triggers somatic embryogenesis in *Theobroma cacao* leaf tissue

**DOI:** 10.1371/journal.pone.0207666

**Published:** 2018-11-26

**Authors:** Andrew S. Fister, Lena Landherr, Melanie Perryman, Yufan Zhang, Mark J. Guiltinan, Siela N. Maximova

**Affiliations:** 1 Pairwise Plants, Durham, NC, United States of America; 2 Department of Plant Science, Pennsylvania State University, University Park, PA, United States of America; 3 Essenlix Corporation, Monmouth Junction, New Jersey; 4 The Huck Institutes of the Life Sciences, The Pennsylvania State University, University Park, PA, United States of America; Kyung Hee Univeristy, REPUBLIC OF KOREA

## Abstract

*Theobroma cacao*, the source of cocoa, is a crop of particular importance in many developing countries. Availability of elite planting material is a limiting factor for increasing productivity of *Theobroma cacao*; therefore, the development of new strategies for clonal propagation is essential to improve farmers’ incomes and to meet increasing global demand for cocoa. To develop a more efficient embryogenesis system for cacao, tissue was transformed with a transgene encoding a fusion of *Leafy Cotyledon 2* (*TcLEC2)* to a glucocorticoid receptor domain (*GR*) to control nuclear localization of the protein. Upon application of the glucocorticoid dexamethasone (dex), downstream targets of LEC2 involved in seed-development were up-regulated and somatic embryos (SEs) were successfully regenerated from *TcLEC2-GR* transgenic flower and leaf tissue in large numbers. Immature SEs regenerated from *TcLEC2-GR* leaves were smaller in size than immature SEs from floral tissue, suggesting a different ontogenetic origin. Additionally, exposure of *TcLEC2-GR* floral explants to dex increased the number of SEs compared to floral explants from control, non-transgenic trees or from *TcLEC2-GR* floral explants not treated with dex. Testing different durations of exposure to dex indicated that a three-day treatment produced optimal embryo regeneration. Leaf derived SEs were successfully grown to maturity, converted into plants, and established in the greenhouse, demonstrating that these embryos are fully developmentally competent. In summary, we demonstrate that regulating TcLEC2 activity offers a powerful new strategy for optimizing somatic embryogenesis pipelines for cacao.

## Introduction

As the source of cocoa beans, *Theobroma cacao* is an important tropical tree crop and a major export from many developing countries. Globally 5–6 million farmers grow cacao, the centerpiece of the multi-billion-dollar chocolate industry. The majority of cocoa comes from low-input, small-holder farms [1]. Production on these farms is significantly hampered by disease [2], decline in soil nutrient content [3, 4] and other biotic and abiotic stressors. As a consequence, effective strategies for germplasm improvement and large-scale vegetative propagation are essential for increasing cocoa production. Efficient propagation systems can help accelerate breeding programs and delivery of new varieties to farmers.

The majority of the world’s cacao supply comes from trees derived from seeds or vegetatively propagated through grafting or making rooted cuttings [3]. These techniques can have several undesirable effects. While farmers often use seeds to expand their farms, doing so often results in loss of desirable traits due to genetic segregation. Grafting or rooting of stem cuttings avoids this problem, but both are labor and cost-intensive, require technical expertise, and often produce plants exhibiting plagiotropism, an undesirable bushy growth habit which lacks a taproot [5]. Propagation of cacao trees through somatic embryogenesis (SE) and tissue culture is emerging as a powerful method for mass tree production [6, 7]. Field evaluation of cacao trees generated through SE has demonstrated yield and bean quality similar to plants generated through traditional propagation methods [8, 9]. An integrated system for carrying out the SE process for propagation of elite varieties leading to distribution of clonal material to farmers has been proposed [6, 7]. A large-scale effort by the Indonesian government used a similar SE-based pipeline for mass propagation of cacao to rehabilitate the country’s cacao production, leading to the generation and distribution to farmers of ~75 million cacao trees which are now productive [6].

While the SE process requires basic laboratory facilities and technical expertise, it is a reliable strategy for fast production of virus- and disease-free plantlets for establishment of clonal stock gardens or direct distribution to farmers. Labs in cacao-producing countries are investing in tissue culture to explore the viability of SE for expanding propagation of elite cultivars (reviewed in [6]). However, the system is genotype-dependent, with some genotypes showing especially poor initiation and conversion rates [5]. Another limitation of SE in cacao is that in over thirty years of cacao tissue culture work, only floral and zygotic embryo tissue have yielded somatic embryos (SEs) that were successfully converted into plants [10]. Also, young cacao trees do not produce flowers until about two years of age, and therefore newly developed varieties could not be used as stock trees for SE until they reach reproductive maturity. Developing a method for somatic embryo production from a previously recalcitrant tissue is an important goal to enable faster propagation of superior cacao varieties.

The study of transcription factors regulating seed and embryo development offers a strategy to modulate the plants’ signal transduction system to speed propagation. Research in other plant systems has described transcription factors including *Leafy Cotyledon (LEC)* family members [11, 12], *Baby Boom* [13], and *Wuschel* [14], key members of evolutionarily conserved signaling networks with roles in seed development [15]. The *LEC* family member *LEC1* was first molecularly characterized in Arabidopsis in a study demonstrating that its overexpression triggered embryo development from vegetative cells [16], and its homolog *LEC2* was shown to have similar function [12]. These proteins have been identified as major regulators of seed storage protein expression [17] and oil synthesis [18], and their potential for engineering plants for optimized biofuel production and SE technology have been explored [19–23].

The inability of cacao leaf explants to form SEs under typical hormonal regimes led us to hypothesize that leaf cells may be lacking some critical component(s) of the signal transduction pathway(s). Therefore, we proceeded to functionally characterize these transcription factors in cacao. We demonstrated that overexpression of *TcLEC2* in transgenic SEs enhanced secondary embryo regeneration, and ectopic expression in leaves activated expression of downstream seed development-related genes [24]. However, we also reported that constitutive overexpression of *TcLEC2* under the control of the CaMV 35S promoter in the stable transgenic SE lines led to spontaneous and recurrent initiation of new embryos that prevented the recovery of mature embryos. Overexpression of cacao *BABY BOOM (TcBBM)* was also shown to enhance embryogenic potential, and resulted in spontaneous embryo development from cotyledons of transgenic embryos [25]. We hypothesized that in order to develop *LEC2*-enhanced SE using leaf explants, a short pulse of *LEC2* expression to induce the embryogenesis pathway was required, but it would be necessary to end this induction to allow embryogenesis and plantlet developmental pathways to proceed normally.

Here, we demonstrate that by regulating the nuclear localization of TcLEC2 with the GR/dex system [26], we were able to regenerate normal primary somatic embryos (PSEs) from leaf explants from a mature transgenic *TcLEC2-GR* expressing tree. After a brief exposure to dexamethasone-containing media, leaf explants produced PSEs that were later converted to plantlets at rates similar to those of PSEs derived from floral explants. *TcLEC2-GR* transgenic floral explants also exhibited enhanced embryo regeneration, elevating productivity beyond previously described rates [27]. These findings advance our understanding of the factors regulating embryogenesis in plants and provide new strategies for enhanced cacao somatic embryogenesis, genetic transformation, and genome editing.

## Material and methods

### *TcLEC2-GR* plasmid construction

The full length *TcLEC2* sequence, without the stop codon, was amplified from pGZ12.0108 (GenBank: KF963132) [24] with SpeI and ApaLI restriction enzyme sites added on 5’ and 3’ end, respectively. The GR domain sequence was amplified from the p35S::LEC2:GR plasmid [18] with a HpaI restriction enzyme site and stop codon added sequentially on 3’ end. Full length *TcLEC2* sequence and *GR* CDS were first assembled in pGEM-T-easy vector (Promega, Madison, WI) using Gibson assembly [28] and proper assembly was verified using Sanger sequencing. Primers for Gibson assembly are listed in Table A in S1 File. *TcLEC2-G*R fusion construct was cloned into pGZ12.0108 using SpeI and HpaI restriction enzyme sites generating pGZ13.0313 (GenBank: KY913902).

### Genomic PCR analysis of transgene insertion

Primers were designed to amplify fragments of *TcLEC2*, *EGFP*, and the vector backbone (pBin19) (Table A in S1 File). Genomic DNA was extracted as previously described using a CTAB extraction protocol [29] from non-transgenic cacao and the transgenic *TcLEC2-GR* tree. Purified plasmid DNA was generated for pGZ13.0313 using Wizard *Plus* SV Minipreps DNA Purification kit (Promega) and diluted to 5 ng/μl with salmon sperm DNA, a 20 μl PCR reactions were setup using Phusion Polymerase (New England BioLabs (NEB), Ipswich, MA) and 5 μM primer (Table A in S1 File) for: 94°C for 2 min, then 35 cycles of 94°C for 30 sec., 50°C for 45 sec, 72°C for 3 min followed by incubation at 72°C for 7 min. In a preliminary experiment, we observed the formation of a heteroduplex dsDNA consisting of the endogenous and transgene *TcLEC2* products that annealed, thus the experiment was repeated with the addition of 5 μl of T7 Endonuclease I (NEB) to each reaction, followed by a 5 min incubation at 37°C to cleave the heteroduplex. Four μls of each PCR reaction were then loaded onto a 1.5% agarose gel (IBI Scientific, Peosta, IA) for electrophoresis.

### Leaf culture initiation and SE production

*Theobroma cacao* stage C leaves from transgenic plants transformed with pGH00.0126 (*35S*::*EGFP* vector control, GenBank: KF018696) pGZ13.0313 (*TcLEC2-GR*), and non-transformed, genotype PSU SCA6 were harvested from plants grown in the greenhouses at Penn State University, University Park, PA [30]. PSU SCA6 is a plant variety maintained at Penn State previously believed to be a clone of the widely used SCA6 cacao genotype but which has been found to be offtype through genotyping analysis. A 5% (v/v) bleach (8.25% sodium hypochlorite solution) was poured into a pre-sterilized 1 liter wide-mouth container 2–3 leaves at a time and gently agitated for 20 minutes. The leaves were rinsed 5x with sterilize distilled-deionized water and then placed on a sterile 150x15mm petri dishes (VWR, Radnor, PA), and cut into 1cm x 1cm sq explants parallel to, but excluding the midvein. Roughly 36–38 explants were generated per leaf selecting only explants with 4 cut edges. Multiple leaves were cut to ensure 12 randomized explants per plate and desired replicate number for each variable. The plant growth regulators (PGRs) Thidiazuron and 2,4-Dichlorophenoxyacetic acid were freshly prepared before making media and added prior to autoclaving. Dex (Sigma-Aldrich, St. Louis, MO, Catalog # D4902) solution was prepared fresh in 100% ethanol and filter sterilized using a sterile syringe filter (Corning, Corning, NY), and added to media after autoclaving. Five dexamethasone (dex) exposure treatments (0hrs, 12hrs, 24 hrs, 3 days, and 1 week) were created by adding dex (10 μM) to PCG in 100x15mm petri dishes. After dex exposure the explants were moved to primary callus growth media (PCG) without dex to complete the 2 wk cycle and cultured for primary SE regeneration as previously described [27]. Mature embryos were counted as described below.

### Flower sterilization, culture initiation and dexamethasone treatment

*Theobroma cacao* unopened, immature flowers were collected from plants grown in the greenhouses at Penn State University, University Park, PA, sterilized and dissected as previously described [27]. A total of 15 flowers from PSU SCA6 trees and 30 flowers from *TcLEC2-GR* transgenic trees were collected. PSU SCA6 flowers were separated into 3 replicates of 5 flowers each and cultured on primary PCG medium. *TcLEC2-GR* flowers were separated into 6 replicates of 5 flowers each, three replicates were cultured on PCG and three on PCG containing 10 uM dex. The cultures were incubated in the dark at 26°C. After 1 week the replicates on PCG + dex were moved to regular PCG. After 2 total weeks culture on PCG all explants were separated into staminodes and petals for each replicate and plated on secondary callus growth (SCG) medium [27]. After 2 weeks culture on SCG all explants were moved to embryo development (ED) medium [27] supplemented with NiSO_4_ (EDN) to a final concentration of 0.005 mg/L. Cultures were transferred to fresh EDN media every 2 weeks.

### Explant preparation for qRT-PCR and histology

Leaf explants from three genotypes (transgenic *TcLEC2-GR*, transgenic pGH00.0126 and non-transgenic PSU SCA6) exposed to four treatments (+/- dex 10 μM and +/- PGR), were sterilized, cut, and plated as described above. After two weeks of culture on PCG, five replicate explants per treatment were frozen in liquid nitrogen to be used for RNA extraction and qRT-PCR analysis.

The remaining replicate explants from all treatments were transferred to SCG for two more weeks, and then were transferred to EDN (refreshed every 2 wks) until the development of PSEs. At 13 weeks ACI, five additional explants from each treatment were fixed overnight at 4°C in 4% Paraformaldehyde (16% Paraformaldehyde, (Electron Microscopy Sciences (EMS), Hatfield, PA) diluted with 0.1M Sodium Cacodylate (EMS). After 24 hrs the samples were loaded in a Leica TP1020 automated tissue processor overnight on the plant tissue setting. The modified Leica program consisted of a series ethanol soaks, 70% for 30 min, 85% for 30 min, 95% for 8 hrs and 30 min, 100% for 2 hrs followed by 3 Histosolve washes for a total of 3 hrs, and 8 hrs in Paraffin wax (Leica Biosystems Surgipath Paraplast Plus, Buffalo Grove, IL). The next day the samples were embedded and mounted using a Leica EG1150H/C. The samples were sectioned using a Shandon Finesse microtome, stained with Hematoxylin and Eosin stain (Leica ST5010 Autostainer XL) and imaged with an Olympus BX61 microscope (Olympus Corporation of the Americas, Center Valley, PA).

### RNA extraction, cDNA synthesis, and qRT-PCR

RNA was extracted from callus tissue using PureLink Plant RNA Reagent (ThermoFisher, Waltham, MA) as previously described [24], and quantified using a Qubit 3.0 spectrophotometer (ThermoFisher). cDNA was synthesized as described [24] using 500 ng of RNA. Gene expression was analyzed as previously described [24] using an ABI StepOne Plus 7300 Realtime PCR System (ThermoFisher) with Takara Premix Ex Taq II SYBR green reagents (Clontech Laboratories, Mountainview, CA). Specificity of primers was evaluated by electrophoresis on a 1% agarose gel stained with GelRed (Biotium, Fremont, CA) and by dissociation curve. Primer sequences for qRT-PCR are listed in Table B in S1 File. Endogenous *TcLEC2* and *TcLEC2-GR* were differentiated by designing primers for the endogenous gene annealing at the 3’ end of the CDS into 3’ UTR, whereas primers for *TcLEC2-GR* annealed at the 3’ end of the *TcLEC2* gene into the CDS of the GR. Relative expression was calculated using the -ΔΔCt method [31] and fold inductions were calculated in REST [32].

### Embryo counts and conversion

For both the leaf and floral explants regeneration experiments, PSEs were counted once they reached 0.5 cm and transferred from EDN to Primary Embryo Conversion (PEC) [33] medium in 100x25 mm petri dishes (ThermoFisher). Due to the smaller size of the embryos, the published protocol was modified to include incubation of the cultures in the dark on PEC for an additional four weeks. Mature embryos were evaluated as normal vs. abnormal embryos as previously described [34]. Embryos with a single well-defined axis and balanced root/shoot symmetry were classified as normal and those fused or without defined cotyledons were classified as abnormal. The abnormal embryos were dissected and cultured for SSE [7]. The normal primary and normal secondary embryos generated from *TcLEC2-GR* leaf explants were taken from PEC medium in the dark and were transferred to fresh PEC and placed in the light. Fifty mature SEs per treatment (5 per plate) were cultured and incubated in the light and number of converted embryos were recorded. Embryos were transferred to fresh medium every 30 days for 3 months at which time embryos with true leaves were considered converted to plants as previously described [7]. The number of embryos that died and the number that had not converted were counted.

### Statistical analysis

For statistical analysis, individual plates of explants were treated as replicates. All tissues and treatments started with three replicates. However, one plate (replicate) from the *TcLEC2-GR* petal explant treatment was lost to contamination. In addition, one of the replicates of the one-week dex exposure treatment of *TcLEC2-GR* leaves did not regenerate SEs and was not included in statistical analysis.

## Results

### Vector construction and production of a *TcLEC2-GR* stably transgenic tree

**Fig 1:**
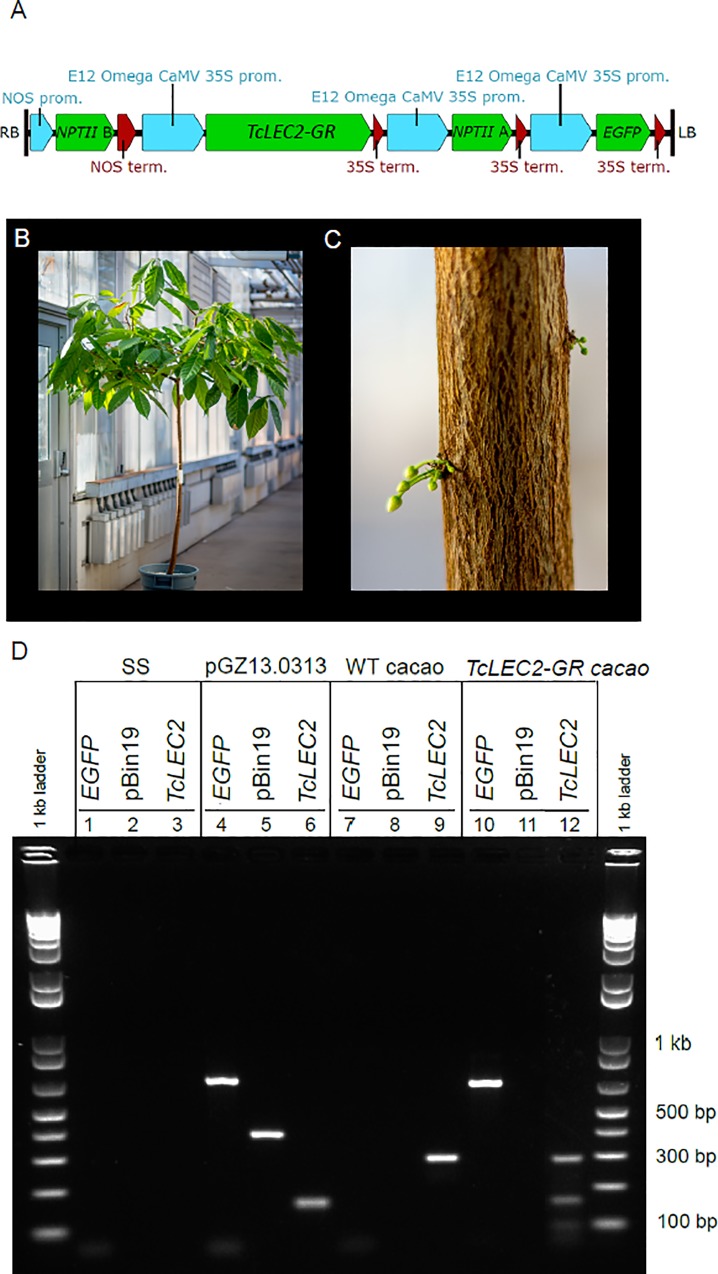
Vector schematic and analysis of stable integration of T-DNA into cacao. **A)** Diagram of the T-DNA region of binary vector pGZ13.0313 containing *TcLEC2-GR* gene. Abbreviations: right and left borders regions (RB and LB), nopaline synthase (NOS), promoter (prom.), terminator (term.). **B)** Photograph of mature *TcLEC2-GR* transgenic cacao tree. **C)** Photograph of unopened flowers developing from the trunk of the TcLEC2-GR transgenic tree. **D)** Genomic PCR analysis of transgenic plants containing *EGFP* and *TcLEC2* transgenes. DNA control and treatment samples are indicated above the gel image: SS—Control salmon sperm DNA; pGZ13.0313—control plasmid DNA; WT—control non-transgenic cacao DNA; and *TcLEC2-GR* cacao—treatment DNA from *TcLEC2-GR* transgenic cacao. PCR amplification was performed using three primer sets: *EGFP* (653 bp amplicon, lanes 1, 4, 7, 10), pBin19 backbone (378 bp amplicon, lanes 2, 5, 8, 11), and *TcLEC2* (288 bp for endogenous gene, 153 bp for transgene, lanes 3, 6, 9, 12). Lane 12 contains *TcLEC2* amplification products from endogenous and transgene versions of *TcLEC2*.

Our prior work demonstrated that constitutive overexpression of *TcLEC2* in cacao tissues resulted in an uncontrolled embryogenic chain reaction [24]. We decided to regulate the effects of TcLEC2 overexpression by fusing the CDS to the glucocorticoid receptor (GR). The GR system has been used in a number of plant systems and is a well-characterized method for controlling the subcellular localization of transcription factors in plant cells [35–38]. After introducing the transgene, TcLEC2-GR product was predicted to remain outside of the nucleus and only translocate into the nucleus upon addition of dex to tissue culture media. We hypothesized that subsequent removal of the embryos from dex-containing media would allow for normal SE development. We designed a binary vector containing a fusion of the *TcLEC2* CDS to the *GR* CDS (pGZ13.0313, GenBank: KY913902, Fig 1A), which was then transformed into *Agrobacterium* strain AGL1.

Following an established cacao genetic transformation protocol [39], cotyledons from secondary SEs were transformed with Agrobacterium containing the *TcLEC2-GR* fusion (pGZ13.0313) and two transgenic embryos were selected based on EGFP expression (green fluorescence). These embryos were grown to maturity and converted into plantlets using a previously described protocol [27, 33], but only one plant was successfully established in the greenhouse. The plantlet was grown to maturity (Fig 1B), and its leaves and flowers (Fig 1C) were subsequently re-introduced to *in vitro* culture to study the effect of regulating LEC2 activity with dex treatment. The mature tree developed normally over several years in the greenhouse with no phenotypic differences compared to non-transgenic trees of the same genotype regenerated from SEs.

To validate insertion and integration of the T-DNA region of pGZ13.0313 into the healthy cacao plant, we performed a genomic PCR analysis as previously described [39], amplifying fragments of EGFP, pBin19 vector backbone, and *TcLEC2* using control vector DNA, control non-transgenic cacao DNA, and DNA extracted from the transgenic cacao plant as templates (Fig 1D). As predicted, *EGFP* bands were only detected in reactions using control vector DNA and *TcLEC2-GR* transgenic cacao (Fig 1D lanes 4 and 10 positive, lane 7 negative). The vector backbone only amplified from control vector DNA (Fig 1D lane 5 positive, lanes 8 and 11 negative), indicating there was no *Agrobacterium* or control plasmid contamination of the wild type or transgenic cacao and that the T-DNA region was transferred from the binary plasmid as expected. Amplification using *TcLEC2* primers produced different sized products depending on the template used. The vector control DNA produced a 153 bp fragment from the *TcLEC2* CDS (lane 6). Amplification from wild type cacao DNA produced a 288 bp band, which includes an intron (lane 9). Amplification using the *TcLEC2-GR* transgenic cacao genomic DNA template resulted in both of the expected two bands, 288 bp from the endogenous gene and 153 bp from the transgene (lane 12), indicating the successful incorporation of the *TcLEC2-GR* fusion into the plant.

### Induction of downstream embryogenic genes by *TcLEC2-GR*

**Fig 2:**
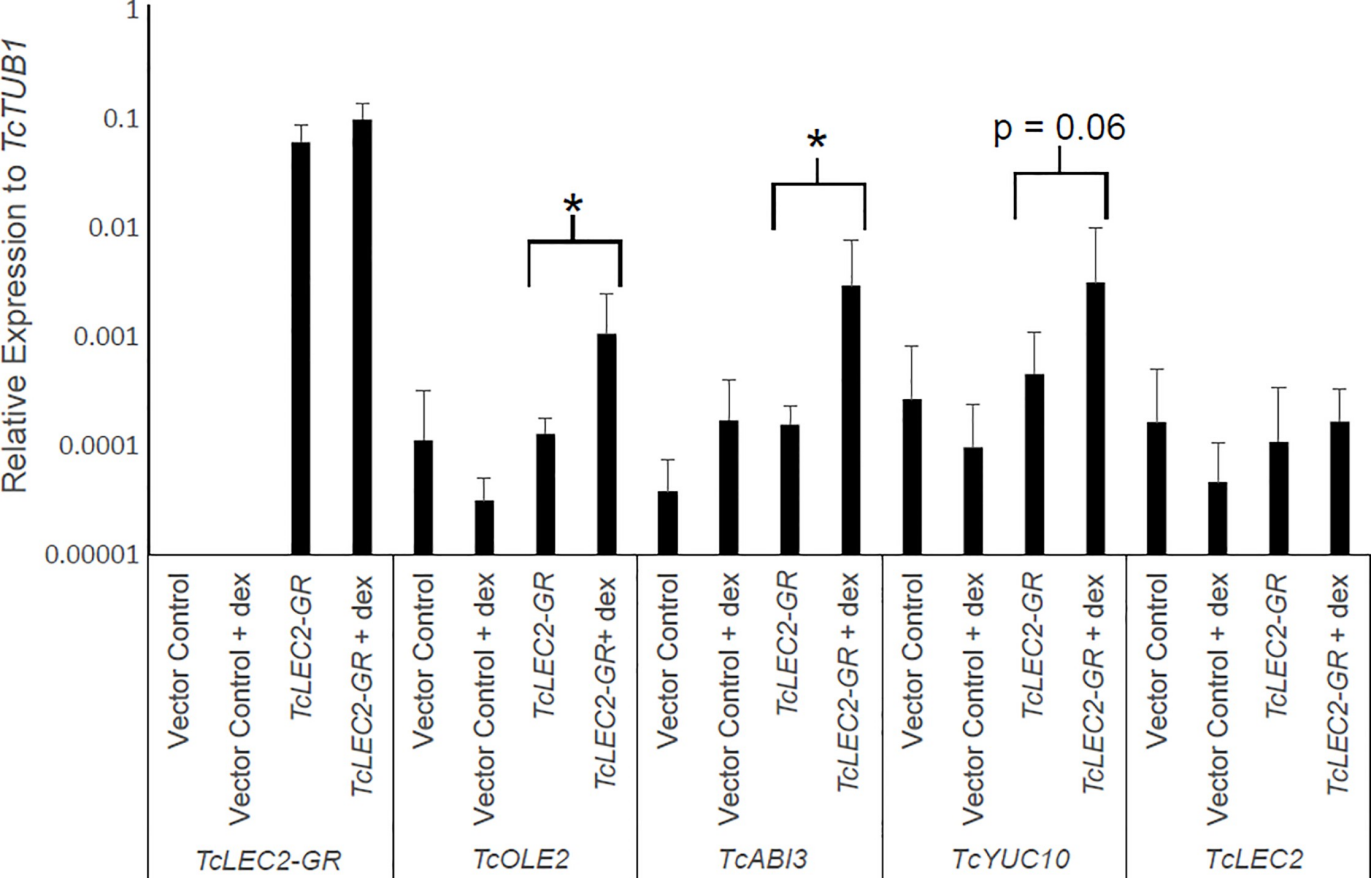
qRT-PCR analysis of *TcLEC2-GR* leaf explants exposed to 10 μM dex for 14 days. Relative expression of embryogenesis-associated transcripts was calculated relative to housekeeping gene *TcTUB1*. Bars represent mean relative expression from five replicates with standard errors. Asterisks denote statistical significance determined using REST software [32].

The ability of *TcLEC2-GR* to induce expression of downstream genes involved in seed development was confirmed by qRT-PCR analysis (Fig 2), confirming prior work in cacao and other plant species [20, 24, 40]. Leaf explants from control transgenic plants transformed with the base vector (pGH00.0126, GenBank: KF018696) and from the *TcLEC2-GR* transgenic plant were cultured for two weeks on PCG media +/- dex. Transcript abundance was measured in callus tissue collected from these cultures using primers designed to amplify a set of candidate TcLEC2 target genes. To allow discrimination of endogenous *TcLEC2* and the *TcLEC2-GR* transgene transcripts, primers specific only to the endogenous *TcLEC2* gene and the *TcLEC2-GR* fusion transgene were used (see Materials and Methods). As expected, endogenous *TcLEC2* was detected in both transgenic and non-transgenic tissues, and the transcript from the *TcLEC2-GR* fusion was detected in only *TcLEC2-GR* transgenic callus. Treatment of the transgenic tissues with dex, allowing nuclear translocation of the overexpressed TcLEC2-GR product, resulted in higher transcript abundance of two downstream seed development regulators, *TcOLE2* and *TcABI3*, 5.5-fold and 8.9-fold respectively (p < 0.05), relative to expression in tissue not treated with dex. A third gene, *TcYUC10* showed 4.9-fold induction with slightly less statistical significance (p = 0.06). Induction of the endogenous *TcLEC2* gene was not detected. Also, no significant effect of the dex treatment on the transcript levels of any of the tested genes was detected in tissue transformed with the vector control. These results demonstrate that as predicted, *TcLEC2-GR* was able to strongly up-regulate specific genes involved in seed development in a dex-dependent manner.

### Embryo regeneration from *TcLEC2-GR* transgenic leaves

**Fig 3:**
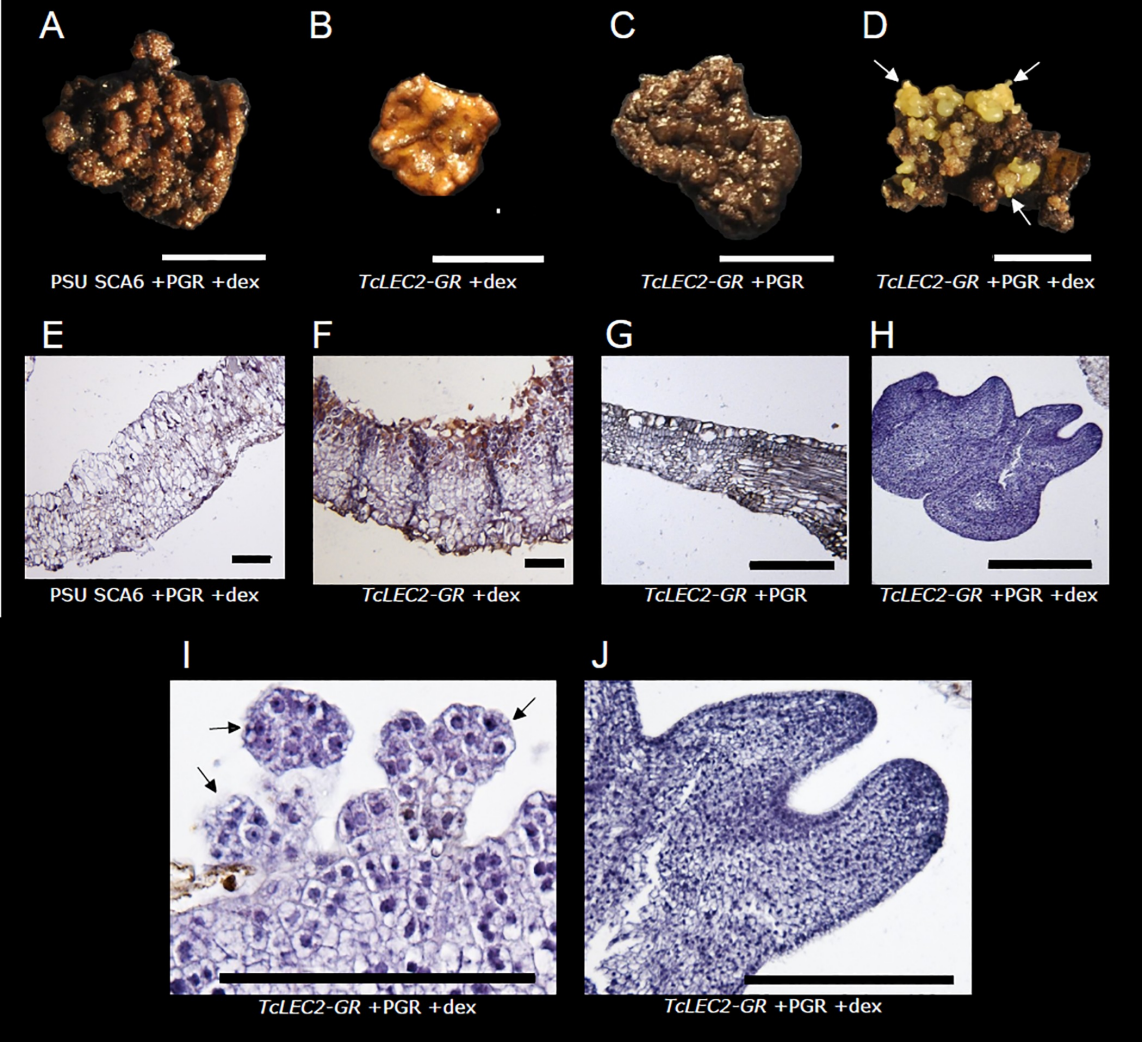
Representative images of somatic embryos regeneration from cacao leaves. **A-D.** Photographs of leaf explants from PSU SCA6 or transgenic *TcLEC2-GR* leaves +/- dex and plant growth regulators (PGR). Arrows in **D** indicate immature embryos. Scale bars = 1 cm. **E-J.** Representative images of histological analysis of leaf explant cross sections from the same treatments as A-D. Scale bars = 0.1 mm. Arrows in (I) indicate nascent globular embryos.

Leaves from the *TcLEC2-GR* plant and a control non-transgenic plant were cultured on media containing dexamethasone (dex) using a standard protocol for induction of SE from floral explants [27]. Leaf explants were sterilized and cultured on PCG medium +/- dex and +/- plant growth regulators (PGRs). Regeneration of SEs was observed only from *TcLEC2-GR* transgenic leaves cultured on medium containing both dex and PGRs (Fig 3A–3D). Callus developed from all other explants treated with PGRs, but no embryo formation was observed. At 13 weeks after culture initiation (ACI), explants were prepared for histological analysis (Fig 3E–3H). Images of explants from control cultures (non-transgenic, + dex, + PGRs) illustrate typical leaf morphology without SE development (Fig 3E–3G), with expected cell density for epidermal and mesophyll layers, and no evidence of embryo formation. Only the *TcLEC2-GR* + dex + PGR tissue (Fig 3H), produced SEs. Leaf SEs at the globular (Fig 3I) and torpedo (Fig 3J) developmental stages showed dense cellular organization previously observed in flower-derived SEs [34].

**Fig 4:**
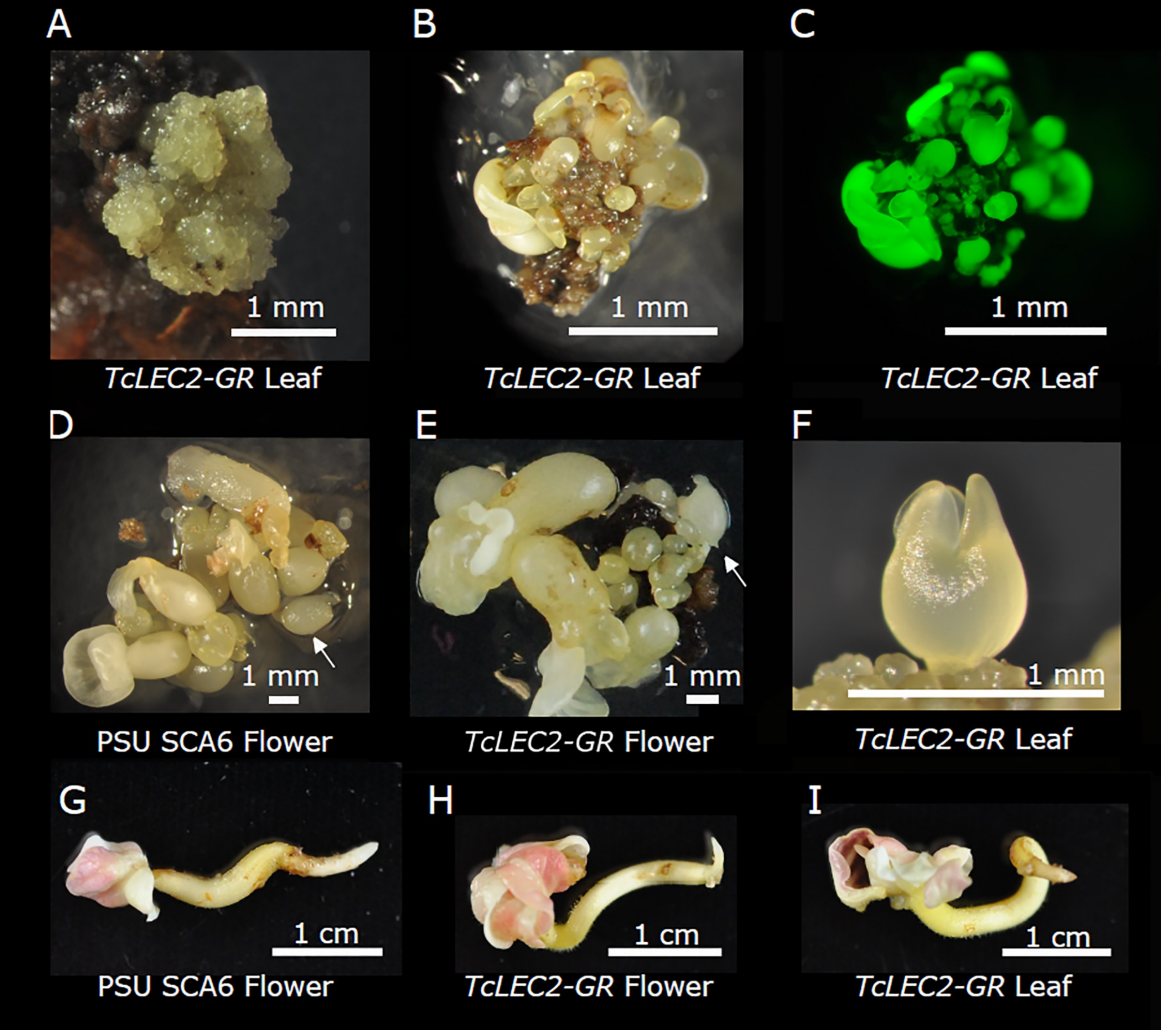
Comparison of embryo maturation from leaf and floral explants. **A.** A cluster of globular embryos developing from *TcLEC2-GR* transgenic callus **B.** Photograph of maturing *TcLEC2-GR* embryo cluster **C.** The same embryo cluster in B photographed to show EGFP fluorescence. **D.** Culture of developing embryos from non-transgenic PSU SCA6 floral tissue and **E.** *TcLEC2-GR* floral tissue. Arrows in D and E indicate early torpedo stage embryos. **F.** An early torpedo embryo from *TcLEC2-GR* leaf callus. **G-I.** Mature somatic embryos derived from non-transgenic floral tissue (G), *TcLEC2-GR* floral tissue (H), and *TcLEC2-GR* leaf tissue (I).

As embryos developed on *TcLEC2-GR* explants, they grew in extremely dense clusters of small globular embryos (Fig 4A). In our previous work, the average diameter of cacao globular embryos derived from a floral explant was >1 mm [34], while the *TcLEC2-GR* leaf-derived globular embryos were ~0.15 mm in diameter. Over time, only ~10% of the embryos in the cluster would continue to develop (Fig 4B and 4C). Compared to non-transgenic floral embryos (Fig 4D) and *TcLEC2-GR* (Fig 4E) transgenic floral embryos, *TcLEC2-GR* leaf embryos were smaller throughout development (Fig 4D–4F). During the cotyledonary stage, however, leaf-derived embryos expanded, reaching the typical size of florally derived SEs by maturity (Fig 4G–4I).

### Dynamics of somatic embryo production from *TcLEC2-GR* flowers and leaves

**Fig 5:**
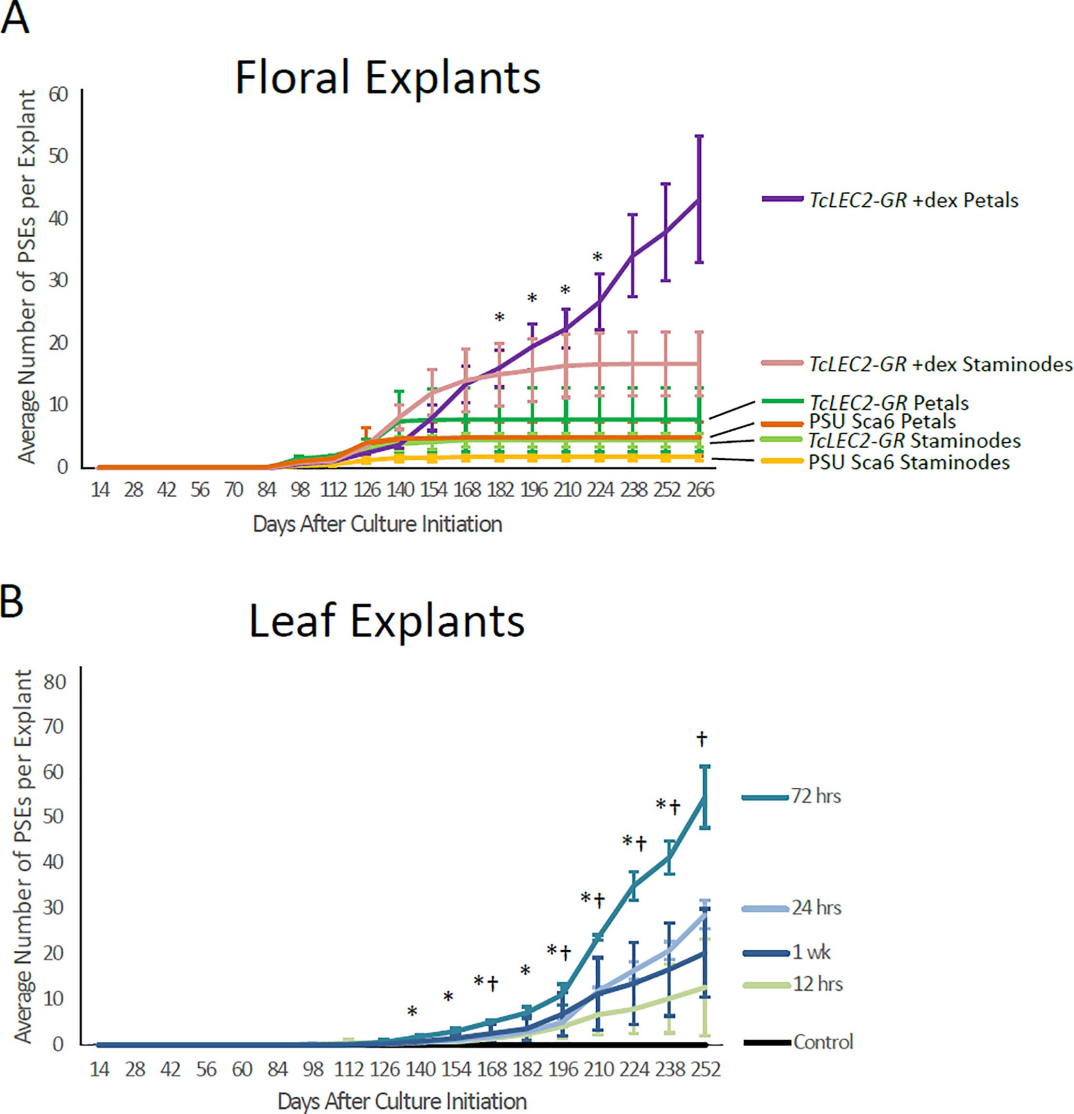
PSE regeneration from transgenic *TcLEC2-GR* floral- and leaf- explants. **A.** Cumulative average of the numbers SEs per explant from floral explants over the time course. Lines represent average numbers of three replicates (two replicates for *TcLEC2-GR* petals after day 126). Error bars represent standard errors. Asterisk denotes statistically significant difference between *TcLEC2-GR* + dex and PSU SCA6 + dex (ANOVA and pairwise t-test p < 0.05). **B.** Cumulative average number of SEs per explant from leaf explants over the time course. Lines represent average number of three replicates (two replicates for 1 wk dex treatment). Error bars represent standard error. Asterisk denotes statistically significant difference (ANOVA and t-test p < 0.05) between 72 hrs treatment and 12 hrs treatment, dagger denotes statistically significant difference between 72 hrs treatment and 24 hrs treatment.

To further explore the dynamics of SE production from *TcLEC2-GR* tissues, we recorded the number of primary SEs (PSEs) regenerated from transgenic *TcLEC2-GR* floral cultures, hypothesizing that regulated *TcLEC2* overexpression would enhance productivity compared to non-transgenic cultures. We measured rates of PSE regeneration from *TcLEC2-GR* petal and staminode explants +/- dex, as well as from control, non-transgenic petal and staminode explants (Fig 5A and Table C in S1 File. In all floral explant cultures, PSEs were first detected at 98 days ACI. PSE regeneration in non-transgenic and non-dex-treated *TcLEC2-GR* cultures continued until 140 days ACI, at which time production ceased, similar to previously reported findings [34]. *TcLEC2-GR* transgenic tissues treated with dex showed a dramatically longer period of productivity compared to other treatments. The *TcLEC2-GR* dex-treated petal tissues generated a significantly higher average (ANOVA and pairwise t test p < 0.05) and total numbers of PSEs, and remarkably, they continued to produce embryos through the duration of the experiment (266 ACI).

A second experiment was conducted to evaluate the response of *TcLEC2-GR* leaf explants to different periods of dex exposure (Fig 5B and Table D in S1 File). PSEs were first detected 98 days ACI as a result of 12- and 24-hr dex treatments. PSEs were first observed at 112 days ACI for 72-hr dex treatment, at 126 days ACI for the 1-wk treatment, and no SEs developed in the no dex treatment. The 72-hr treatment was ultimately the most productive, with significantly more PSEs than the 12 and/or 24-hr treatments throughout the majority of the time course (ANOVA and pairwise t test p < 0.05). (Table D in S1 File). Ultimately, *TcLEC2-GR* leaf explants showed the capacity for embryo regeneration at rates similar to those achieved with floral explants.

### Somatic embryo quality

A basic feature of the cacao floral explant SE system is the appearance of a mixture of normal and abnormal PSEs, with a large fraction of embryos showing physiological abnormalities at various developmental stages. The proportion of normal embryos can be used as an index of Embryo Quality (EQ). EQ has been measured in our floral SE system in the past and ranges from ~14% for primary embryogenesis in staminode explants to between 40% and 80% for secondary embryogenesis initiated from primary SE cotyledon explants [33, 34, 41]. We measured EQ of PSEs regenerated from *TcLEC2-GR* floral and leaf explants. Consistent with our previous results, a small fraction of PSEs from floral explants were normal (11–15%, Table C in S1 File). The percentage of normal PSEs regenerated from leaves was slightly higher at 15–20% (Table D in S1 File), but still markedly lower than previous measurements for secondary SEs (SSEs) [33, 41].

### Conversion of leaf-derived somatic embryos

**Fig 6:**
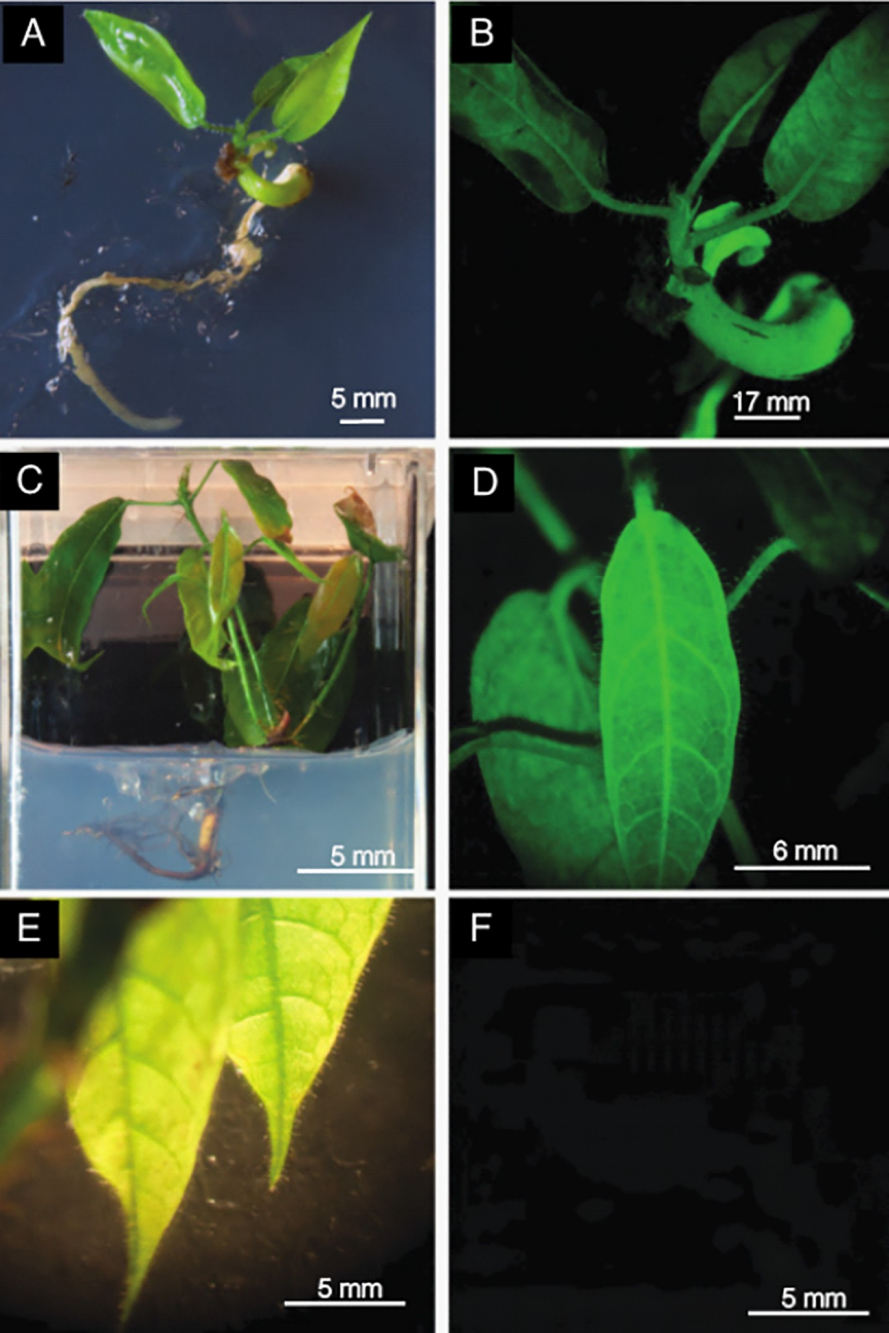
Green fluorescent protein expression imaging of SE-derived plantlets regenerated from transgenic *TcLEC2-GR* leaf explants. **A.** Light Image of mature somatic embryo in conversion. **B.** Fluorescent image of the same embryo (A), showing strong expression of EGFP. **C.** Light image of *TcLEC2-GR* plantlet **D.** Fluorescent image of same plantlet (C), expressing *EGFP*. **E.** Light image of control non-transgenic plantlet similar in developmental state to plantlet in C, regenerated from PSU SCA6 cacao flower. **F.** Fluorescent image of same embryo (E), lacking green fluorescence. Fluorescence imaging was performed with exposure times of 8 sec. for B and 20 sec. for images D and F. For clarity, the brightness was increased equally for images D and F using Adobe Photoshop brightness setting 100.

The process of developing a mature somatic embryo into a plantlet is termed embryo conversion. Another measure of embryo viability is the percentage of SEs which successfully convert, which we measured in the *TcLEC2-GR* leaf-derived cultures. In two experiments, normal PSEs regenerated from *TcLEC2-GR* leaves had conversion rates of 30% and 47.4%, similar to previous measurements of PSE conversion [39] (Table E in S1 File). Cotyledons of PSEs regenerated from leaves were used as explants to generate SSEs, which converted at a higher rate of 63.4%, also similar to previously reported conversion rates for SSEs from cotyledon explants of PSEs derived from flowers [42]. Plantlets derived from *TcLEC2-GR* leaf-derived embryos grew normally (Fig 6A, 6C and 6E light images). Fluorescence microscopy was used to image EGFP expression in the converted transgenic and control plants. As expected, we observed EGFP expression in leaves of converted *TcLEC2-GR* plantlets (Fig 6B) and control empty vector transgenic plantlet (Fig 6D), but not in the control non-transgenic SE (black image in Fig 6F).

## Discussion

In this study, we demonstrate that overexpression of *TcLEC2* fused to the glucocorticoid receptor domain (*TcLEC2-GR*) is sufficient to induce SE in cacao leaves in a dex-dependent manner. PSEs regenerated from leaf explants after removal of dex from the culture media exhibited small initial size, but reached maturity, successfully converted to plantlets, and acclimated to greenhouse conditions at rates similar to those seen for PSEs from floral explants [34]. It is remarkable that cacao leaf tissue explants are not competent for SE induction, but the addition of a single protein, along with a suitable hormone regimen, is sufficient to reprogram leaf tissue to initiate the SE pathway. *TcLEC2-GR* floral explants were also more productive than control non-transgenic tissue after initial dex treatment, further demonstrating the importance of *TcLEC2* in the embryogenesis regulatory network. Optimizaton of our dex treatment may improve SE in floral and leaf explants. High concentrations of dex can be toxic to plant tissue [43], which may explain the lower number of PSEs per leaf explant in our 1 week dex treatment. Future work could focus on assaying a narrower range of dex concentrations.

Our previous analysis of *TcLEC2* used a *35S*::*TcLEC2* transgene without GR/dex control, and resulted in an “embryogenic chain reaction,” wherein it was not possible to recover mature embryos. Here, fusing *TcLEC2* to *GR* and using a short exposure to dex induced embryogenesis in the cultures, enabled embryos to proceed through development, maturation, and conversion pathways, and become acclimated as greenhouse-grown plants. We did not show direct evidence of translocation of the fusion protein into the nucleus upon dex treatment, although this mechanism has been described in a variety of plants [43–45]. However we provide evidence for the activity of our fusion protein by measuring the induction of genes known to be downstream targets of *TcLEC2* (*TcABI3* [46] and *TcOLE2* [18]) in leaf-derived *TcLEC2-GR* callus exposed to dex. We also recorded an induction with marginal significance of *TcYUC10*, which supports previous reports that YUC proteins, involved in auxin biosynthesis, act downstream of LEC2 and are involved in the promotion of embryogenesis [40].

Epigenetic factors also contribute to SE, with low methylation correlating to embryogenic capacity [47–49].The Polycomb repressive complex 2 (PRC2) was recently shown to suppress hormone-induced somatic embryogenesis in Arabidopsis through epigenetic modification of pathways involved in totipotency [50]. DNA demethylation was also demonstrated to co-occur with auxin synthesis during induction of somatic embryogenesis from leaf cultures in white oak [51]. Recalcitrant tissues like cacao leaves may feature chromatin modifications which prevent hormone-mediated reprogramming of tissue *in vitro*. However, ectopic expression of developmental regulators like WUSCHEL [15], BABY BOOM [13, 15, 25], LEC1 [21], and LEC2 [21, 40] may bypass heterochromatin-based repression, enabling reprogramming in recalcitrant tissues and enhancing embryogenic capacity in tissues for which SE is already possible. Differences in chromatin state and the resulting differences in gene expression may also contribute to genotype-to-genotype variability in SE efficiency. In cacao, SE efficiency in floral explants varies across accessions representing several of cacao’s genetic groups [6, 34, 41, 52]. Analysis of methylation and transcriptomic differences between floral explants of these accessions could explain variability observed in SE and clarify the epigenetic or gene expression differences between tissues which result in cacao leaf recalcitrance. Embryos developing from cacao leaf explants were initially smaller than those in floral explants, suggesting that ectopic TcLEC2 expression may not completely alleviate epigenetic or transcriptional barriers to early embryo development in leaf tissue, however our use of the TcLEC2-GR was ultimately sufficient to recover physiologically normal mature somatic embryos. Further analysis will also be required to demonstrate that the *TcLEC2-GR* system is sufficient for induction of embryogenesis in leaf explants from other cacao accessions.

Our experiments focused on regeneration of primary SEs from leaf and floral explants, however secondary and tertiary embryogenesis are often used for propagation and show greater average numbers of embryos regenerated per explant and increased rates of embryo conversion. Previous reports of PSE regeneration from floral explants range from about 4–45 embryos per explant depending on the genotype, and up to ~70 embryos per explant in secondary embryogenesis [34]. PSU SCA6, the genotype which performed best in that study, has become a model variety for optimizing SE in cacao, and is the same genotype used in the present work. Here, dex-treated *TcLEC2-GR* cacao produced ~50 PSE embryos per explant from petals and leaves at the end of a ~250-day time course. We also observed a slight, non-statistically significant increase in embryo production in *TcLEC2-GR* floral explants without dex treatment relative to PSU SCA6 floral explants, suggesting that some leakiness in the GR regulation may subtly enhance embryogenesis. Future work will determine whether the *TcLEC2-GR* system can also enhance regeneration in secondary and tertiary somatic embryogenesis. Compared to previous reports, expression of the transgene did not affect the proportion of normal and abnormal primary embryos regenerated from leaves, nor was there a decrease in rate of embryo conversion [33, 39, 41, 42].Further analysis is also needed to compare the proportion of normal and abnormal secondary SEs regenerated from primary leaf-derived SEs [6, 34, 42].

The transgenic plant material described in this manuscript was provided to an independent group that published a preliminary set of experiments evaluating *TcLEC2-GR* expression [53]. While the published manuscript corroborates some of our findings, it contains results that are not reproducible. One of the major results reported is the observation of newly regenerated tertiary SEs as early as six days ACI in a single experiment using the regeneration protocol developed by our group. In our experience with cacao tissue culture since 1998, cacao globular SEs have never been observed in a time frame of less than 4 weeks with or without *TcLEC2* overexpression. In fact, given the roughly two-day cell division rate of cacao cells in culture, it is literally impossible for visible globular embryos to develop in only 6 days. Nonetheless, we repeated the described experiment with multiple replicates and we did not observe embryos at 6 days ACI. We speculate that the depicted outgrowths in Fig 4 of Shires et al. are misidentified as SEs and could be micro-calli formed during the first week after culture initiation. Another possibility is that the observed “new globular embryos” were formed during prior regeneration events and were carried over in the secondary SE tissue, although we can only speculate because a detailed description of the tissue used for the experiment is lacking. Additionally, the report by Shires et al. includes other omissions and errors in the methodology that make their qRT-PCR and dex dose-response experiment results impossible to validate.

In our previous study, constitutive overexpression of *TcLEC2* triggered an embryogenic chain reaction that prevented recovery of normal mature somatic embryos [24]. In this study we demonstrate that regulating *TcLEC2* subcellular localization using the GR/dex system induces genes involved in regulating seed development, produces normal embryos, boosts embryo production in floral explants and enables regeneration of embryos from leaves. A remaining limitation of the method is the reliance on the *TcLEC2-GR* transgene to boost embryogenic capacity. While this technique is immediately useful for improving SE, further work is required to develop a transgene-free system for leaf explant SE in cacao. Nonetheless, the method holds potential for the enhancement of genetic transformation, genomic editing, and regeneration of cacao somatic embryos in the lab. Future work investigating epigenetic and transcriptional barriers to tissue reprogramming in cacao may lead to development of a system for improved in SE in non-transgenic cacao plants.

## GenBank numbers

**pGZ13.0313** *35S*::*TcLEC2-GR* binary vector, GenBank: KY913902

**pGH00.0126** *35S*::*EGFP* binary vector, GenBank KF018696

**pGZ12.0108** *35S*::*TcLEC2* binary vector, GenBank: KF963132

## Supporting information

S1 File Tables of additional data including: primer sequences for validation of *TcLEC2-GR* transgenic plant and qRT-PCR (Tables A and B), average number of embryos regenerated from floral and leaf explant experiments (Tables C and D), embryo conversion data (Table E).
(DOCX)
