# Supplementary material for: Glucocorticoid receptor-regulated TcLEC2 expression triggers somatic embryogenesis in Theobroma cacao leaf tissue
Source: PLoS One. 2018 Nov 26;13(11):e0207666. doi: 10.1371/journal.pone.0207666 (PMC6261025; doi:10.1371/journal.pone.0207666)
Supplement: S1 File — Tables of additional data including: primer sequences for validation of TcLEC2-GR transgenic plant and qRT-PCR (Tables A and B), average number of embryos regenerated from floral and leaf explant experiments (Tables C and D), embryo conversion data (Table E). (DOCX) [file pone.0207666.s001.docx]

| **Region Amplified** | **Amplicon (bp)** | **Forward Primer Sequence** | **Reverse Primer Sequence** |
| --- | --- | --- | --- |
| *TcLEC2* (Gibson) | 1,300 | 5’-ccgcgggaattcgattACTAGTATGGGTTGGTCTC-3’ | 5’- tttcgagcGTGCACAAGTGAAAAATTG-3’ |
| GR (Gibson) | 863 | 5’-ttgtgcacGCTCGAAAAACAAAGAAA-3’ | 5’-gcgaattcactagtgattGTTAACTCATTTTTGATGAAAC-3’ |
| Bin19 Backbone | 378 | 5’-GGCTGTCGATCTTGAGAACTATG-3’ | 5’-CTGCTCGTTCCAGTAGCTTTAG-3’ |
| *EGFP* | 653 | 5’-GTAAACGGCCACAAGTTCAGCGT-3’ | 5’-TTACTTGTACAGCTCGTCCATGCC-3’ |
| *TcLEC2* fragment | CDS = 153 Endogenous = 288 | 5’-TGACCAGCTCTGGTGCTGACAATA-3’ | 5’-ATTCTCCCAAGGGACCCAACATCA-3’ |

**Table A.** Primer sequences for plasmid construction and for analysis of *TcLEC2-GR* stable integration.

**Table B.** Primers sequences for qRT-PCR analysis of downstream embryogenic genes.

| **Primer ID** | **Sequence** |
| --- | --- |
| *TcTUB1* qRT F | GGAGGAGTCTCTATAAGCTTGCAGTTGG |
| *TcTUB1* qRT R | ACATAAGCATAGCCAGCTAGAGCCAG |
| *TcLEC2+GR* qRT F | TGCTATGGCGGTCTTGATATG |
| *TcLEC2+GR* qRT R | GAAGTGTCTTGTGAGACTCCTG |
| *TcOLE2* qRT F | CACACTGGGTGCAACGCTG |
| *TcOLE2* qRT R | GGCTGAAAATGACCATGAGTGGCG |
| *TcABI3* qRT F | AAACCCTACCTCGAACCCAAACCT |
| *TcABI3* qRT R | ATCCAGGGTGGCTGAGAGAAACAA |
| *TcYUC10* qRT F | CGAAGCAAGGAACATGCAATC |
| *TcYUC10* qRT R | GGAAGGTCAGGAATGTACTTGG |
| *TcLEC2* 3' qRT F | TGCTATGGCGGTCTTGATATG |

**Table C.** Total numbers of mature primary somatic embryos (PSEs) generated from *TcLEC2-GR* and non-transgenic floral tissue. Averages (Ave) represent mean across three replicates and SE denotes standard error calculated from those replicates.

| **Treatment (genotype: tissue explant)** | **Total PSEs**  **(Ave # ± SE)** | **Normal PSEs (Ave # ± SE)** | **Abnormal PSEs (Ave # ± SE)** | **Normal PSEs**  **(% ± SE)** |
| --- | --- | --- | --- | --- |
| PSUSCA6: Petals | 64.00 ± 25.9 | 7.33 ± 2.96 | 56.67 ± 22.93 | 11.54 ± 0.26 |
| PSUSCA6: Staminodes | 42.00 ± 13.6 | 4.67 ± 1.45 | 37.33 ± 12.25 | 11.60 ± 1.08 |
| *TcLEC2-GR*: Petals | 41.67 ± 13.0 | 4.00 ± 0.58 | 37.67 ± 12.44 | 10.90 ± 2.16 |
| *TcLEC2-GR*: Staminodes | 130.67 ± 36.99 | 14.00 ± 3.22 | 116.67 ± 34.53 | 11.46 ± 1.68 |
| *TcLEC2-GR+dex*: Petals | 496.33 ± 44.92 | 74.33 ± 8.45 | 422.00 ± 40.53 | 15.03 ± 1.31 |
| *TcLEC2-GR*+dex: Staminodes | 195.67 ± 72.17 | 23.33 ± 7.22 | 172.33 ± 64.95 | 12.83 ± 1.34 |

**Table D.** Total numbers of primary mature somatic embryos (SE) generated from *TcLEC2-GR* transgenic leaf explants after exposure to dex for different periods of time. Average (Ave) and standard error (SE) are calculated from three replicates, except for the 1 wk treatment, which is based on only two replicates.

| **Treatment** | **Total PSEs**  **(Ave # ± SE)** | **Normal PSEs**  **(Ave # ± SE)** | **Abnormal PSEs**  **(Ave # ± SE)** | **Normal SEs**  **(% ± SE)** |
| --- | --- | --- | --- | --- |
| 0 hours | 0 | 0 | 0 | 0 |
| 12 hours | 149.67 ± 75.30 | 27.00 ± 12.06 | 122.67 ± 63.28 | 19.66 ± 2.43 |
| 24 hours | 345.67 ± 21.26 | 52.33 ± 8.45 | 293.33 ± 13.38 | 14.97 ± 1.56 |
| 3 days | 581.33 ± 56.57 | 88.00 ± 8.51 | 493.33 ± 49.01 | 15.17 ± 0.71 |
| 1 week | 243.50 ± 82.50 | 35.00 ± 13.00 | 208.50 ± 69.50 | 14.19 ± 0.53 |

**Table E.** Number of *TcLEC2-GR* transgenic embryos (SE) converted to plants.

| **Experiment 1** |  |  |  |  |  |
| --- | --- | --- | --- | --- | --- |
| **Treatment** | **Total # SE** | **# SE Converted** | **# SE Dead** | **# SE Not Converted** | **Conversion Rate** |
| *TcLEC2-GR* leaves +dex, primary SE | 100 | 30 | 21 | 49 | 30% |
| **Experiment 2** |  |  |  |  |  |
| **Treatment** | **Total # SE** | **# SE Converted** | **# SE Dead** | **# SE Not Converted** | **Conversion Rate** |
| *TcLEC2-GR* leaves +dex, primary SE | 116 | 55 | 41 | 20 | 47.0% |
| *TcLEC2-GR* leaves +dex, secondary SE | 41 | 26 | 2 | 13 | 63.4% |
| *TcLEC2-GR* flowers +dex, secondary SE | 85 | 40 | 20 | 25 | 47.1% |
